# Prognostic Value of Coronary Flow Reserve Obtained on Dobutamine Stress Echocardiography and its Correlation with Target Heart Rate

**DOI:** 10.5935/abc.20170041

**Published:** 2017-05

**Authors:** José Sebastião de Abreu, Eduardo Arrais Rocha, Isadora Sucupira Machado, Isabelle O. Parahyba, Thais Brito Rocha, Fernando José Villar Nogueira Paes, Tereza Cristina Pinheiro Diogenes, Marília Esther Benevides de Abreu, Ana Gardenia Liberato Ponte Farias, Marcia Maria Carneiro, José Nogueira Paes Junior

**Affiliations:** 1 Clínica Clinicárdio, de Fortaleza, CE - Brazil; 2 Hospital Prontocárdio, Fortaleza, CE - Brazil; 3 Hospital das Clínicas da Universidade Federal do Ceará, Fortaleza, CE - Brazil

**Keywords:** Echocardiography, Stress, Heart Rate, Prognosis, Fractional Flow Reserve, Myocardial

## Abstract

**Background:**

Normal coronary flow velocity reserve (CFVR) (≥ 2) obtained in the left anterior descending coronary artery (LAD) from transthoracic echocardiography is associated with a good prognosis, but there is no study correlating CFVR with submaximal target heart rate (HR).

**Objective:**

To evaluate the prognostic value of CFVR obtained in the LAD of patients with preserved (>50%) left ventricular ejection fraction (LVEF) who completed a dobutamine stress echocardiography (DSE), considering target HR.

**Methods:**

Prospective study of patients with preserved LVEF and CFVR obtained in the LAD who completed DSE. In Group I (GI = 31), normal CFVR was obtained before achieving target HR, and, in Group II (GII = 28), after that. Group III (G III=24) reached target HR, but CFVR was abnormal. Death, acute coronary insufficiency, coronary intervention, coronary angiography without further intervention, and hospitalization were considered events.

**Results:**

In 28 ± 4 months, there were 18 (21.6%) events: 6% (2/31) in GI, 18% (5/28) in GII, and 46% (11/24) in GIII. There were 4 (4.8%) deaths, 6 (7.2%) coronary interventions and 8 (9.6%) coronary angiographies without further intervention. In event-free survival by regression analysis, GIII had more events than GI (p < 0.001) and GII (p < 0.045), with no difference between GI and GII (p = 0.160). After adjustment, the only difference was between GIII and GI (p = 0.012).

**Conclusion:**

In patients with preserved LVEF and who completed their DSE, normal CFVR obtained before achieving target HR was associated with better prognosis.

## Introduction

For decades stress echocardiography has been used to assess coronary artery disease (CAD), and has been established as an important diagnostic and prognostic tool.^1-3^ The most used pharmacological stressors are those that act as vasodilators (dipyridamole and adenosine) or those that increase myocardial oxygen consumption (dobutamine) by increasing cardiac work.^4^ However, the literature shows that, in addition to the consistent positive inotropic effect, the action of dobutamine as a coronary vasodilator might provide important information during dobutamine stress echocardiography (DSE).^5,6^

The assessment of coronary flow velocity reserve (CFVR) in the left anterior descending coronary artery (LAD) has been validated, and this noninvasive measurement has been often used in the clinical setting, because it adds diagnostic and prognostic value to pharmacologic stress echocardiography.^7-15^ Despite their distinct mechanisms of action, the myocardial flow responses to adenosine and dobutamine in CAD have a linear correlation, dobutamine being comparable to adenosine in the same population with preserved left ventricular ejection fraction (LVEF), and both drugs provide concordant CFVR values.^5,6^

Several publications have considered a CFVR value ≥ 2 as normal and suitable to infer good prognosis or absence of significant coronary artery stenosis.^6,10-12,16-20^ When CFVR values are higher at the early stages of DSE, the exam is expected to be completed with no contractile abnormality compatible with myocardial ischemia.^21^ However, a low CFVR value at the early stages of DSE can anticipate the occurrence of myocardial ischemia manifest as contractile abnormality.^22^

Normal CFVR in the LAD can be obtained before (early) or after (late) submaximal target heart rate (HR) is reached.^20,23^ Although the relevance of CFVR has been established, the meaning of normal CFVR obtained at the early stage of DSE has not been clarified. Thus, this study aimed at assessing the prognostic value of CFVR obtained in the LAD of patients with preserved LVEF (>50%) who completed DSE after reaching submaximal target HR.

## Methods

This is a prospective observational study performed during two years in a population selected from the previous study by Abreu et al.,^23^ which has assessed CFVR during DSE.

The decision to refer patients with known or probable CAD for assessment with DSE was exclusively up to their attending physicians. After collecting the clinical history, risk factors for CAD were assessed and transthoracic echocardiography was performed. When not contraindicated, patients underwent DSE. The exclusion criteria were as follows: uncontrolled arterial hypertension; unstable angina; congestive heart failure; recent myocardial infarction (within one month from DSE); important heart valvular disease; prostate disease or glaucoma with contraindication for atropine use; and non-sinus rhythm.

The present study included patients with preserved LVEF (>50%) on transthoracic echocardiography and who completed DSE after attaining submaximal target HR. Normal CFVR (≥ 2) was classified into early or late, based on being obtained before or only after reaching submaximal HR, respectively. In all cases with abnormal CFVR values, CFVR recording was obtained at the end of DSE. The DSE protocols and CFVR recordings are described below.

### Dobutamine stress echocardiography

At our service, the DSE protocol instructs patients to suspend beta-blockers 72 hours before the exam, and to resume their use after the procedure. The other drugs should be maintained. All patients were informed about the risks and objectives of the exam, which was only initiated after the patient's verbal consent. For the DSE, the Vivid 7 echocardiography device (GE Healthcare) with second harmonic image and the M4S multifrequency transducer with frequency ranging from 2 to 4 MHz were used. The left ventricle was visualized in the apical (4- and 2-chamber) and parasternal (long and short axes) views at rest and during dobutamine use at the doses of 10 (low dose), 20, 30 up to 40 *µ*g/kg/min and 3-minute intervals. The images were obtained at rest, low-dose, peak and recovery phases, and compared on a quadruple screen. Atropine could be added after the second stage (incremental doses of 0.25 mg up to the maximal cumulative dose of 2 mg). The DSE was completed after submaximal target HR [(220 − age) x 85%] was attained and/or myocardial ischemia was found.

Ischemia was considered as the report of typical angina, new contractile abnormality or worsening of a preexisting one (except from akinesia to dyskinesia). The exam would be interrupted in the presence of intolerance to medication, hypertensive peak (blood pressure > 230/120 mm Hg), or cardiac arrhythmia. The left ventricle was divided into 16 segments, and a numerical score was given to each segment depending on contractility as follows: normal = 1; hypokinetic = 2; akinetic = 3; or dyskinetic = 4. The segmental contraction score index was calculated by dividing the points obtained by 16.^1,2,23^

### Left anterior descending coronary artery assessment

Pulsed color Doppler of the LAD was recorded in the left lateral decubitus, the same position used for the DSE. The LAD was visualized in its mid-distal region with a pre-established specific preset, based on acquisition from the low parasternal long-axis, 2-chamber or modified 3-chamber view, concomitant with little adjustments of angulation or rotation of the transducer. Using a small box of color Doppler with Nyquist limit of approximately 20 cm/s, LAD appeared as a tubular image, in which the greatest possible stretching and extension were determined, as well as the smallest angulation, with the Doppler cursor, whose sample volume was 2 mm. By use of pulsed Doppler, the flow assessed was characterized by biphasic spectrum with diastolic predominance, and anterograde curves above baseline were recorded. Initially, the Doppler velocity scale was limited to 80 cm/s and could be widened during DSE, allowing the capture of subsequent velocity increases of the Doppler curves.

By using Doppler assessment of LAD synchronized with electrocardiography, peak diastolic velocities (PDV) were recorded, selecting three spectral curves at rest and during stress, not necessarily continuous, but with good quality and higher velocities. The CFVR was obtained by dividing the PDV (mean of three peaks) occurring during DSE by the baseline PDV (mean of three peaks) recorded at rest. By use of the same transducer, visualization of the two-dimensional left ventricular image and of Doppler of the LAD was alternated. Thus, the quadruple screen of the DSE was filled in the different stages, concomitantly with PDV recordings until the end of the exam. Right after completing the exam, the DSE result was defined, and CFVR, calculated.^6,18-20,22-24^

During the study, the patients' management was determined exclusively by their attending physicians. Independently of their groups, the patients were followed up to assess the occurrence of events, which were established as follows: cardiovascular death; acute coronary insufficiency; coronary intervention (hemodynamic or surgical); coronary angiography (without further intervention during follow-up); and hospitalization (due to angina pectoris, heart failure, or cardiac arrhythmia). Cardiovascular death was considered as death secondary to any of the events cited or any other condition with acute cardiac impairment. Because of the different intensities and possible gradation of events, in the absence of death, the follow-up of all patients was maintained.

Information on all patients' clinical outcome was obtained through contact with the patients, their guardians or attending physicians, and through medical record or death certificate review.

### Statistical analysis

Continuous variables were expressed as mean ± standard deviation, while categorical variables were expressed as absolute number and percentage.

Data descriptive analysis per group was performed by use of contingency tables and descriptive measures. The homogeneity of the groups in regard to the categorized variables was tested by use of Fisher exact test. The normality of the distribution of quantitative variables per group was assessed by use of Shapiro-Wilk test. The homogeneity of the groups regarding variances was assessed with Levene test. The homogeneity of the groups regarding quantitative variables was analyzed with ANOVA (analysis of variance) for variables with normal distribution, or with nonparametric Kruskal-Wallis test of independent samples for variables with non-normal distribution. The variables whose groups differed significantly underwent sub-hypothesis tests by use of the minimum significant difference test. Overall survival for the event in the groups was analyzed by use of Kaplan-Meier regression. The groups were adjusted by use of Cox regression and Wald statistics, and underwent pairwise comparison. The analyses were performed with the SPSS software 20.0 (SPSS Inc., Chicago, IL, USA). For all analyses, p < 0.05 was adopted as statistically significant.

## Results

### Clinical characteristics

Of the 100 patients with LAD flow obtained at rest, 92 could have their LAD flow obtained during stress. However, 5 patients could not complete their DSE. Therefore, this study population consisted of an initial sample of 87 patients.

The assessment lasted 28 ± 4 months, and follow-up was performed in 83 patients of the 87 (95.4%), because 1 patient with early CFVR and 3 with abnormal CFVR were lost to follow-up. Of the 59 patients with normal CFVR, 31 had early CFVR (Group I) and 28 had late CFVR (Group II). Group III consisted of 24 patients with abnormal CFVR. The clinical data of the 83 patients studied were as follows: mean age, 63 ± 11 years; men, 48 (57.8%); hypertensive, 58 (70%); dyslipidemic, 53 (64%); diabetic, 12 (14.5%); and known CAD, 24 (29%) patients. Table 1 shows that those clinical data did not differ between the groups, and neither did the body mass index. Regarding medications, the analysis of homogeneity between the groups did not differ concerning the use of the following drugs: antiplatelet agents (p = 0.059); anti-hypertensive agents (p = 0.924); lipid-lowering drugs (p = 0.257); hypoglycemic agents (p = 0.792); and nitrates (p = 1.000). The time elapsed between DSE and an event occurrence did not differ between the groups. The event occurrence, however, differed.

**Table 1:** Clinical aspects

	Group I	Group II	Group III	p
Patients	31 (100)	28 (100)	24 (100)	
Age (years)	60 ± 10	64 ± 12	66 ± 8	0.092
Women	13 (42)	9 (32)	13 (54)	0.273
BMI (kg/m^2^)	27.5 ± 4.5	27.3 ± 3	28.5 ± 7	0.991
Hypertension	18 (58)	21 (75)	19 (79)	0.216
Dyslipidemia	19 (61)	19 (68)	15(62.5)	0.881
Diabetes	5 (16)	4 (14)	3 (12.5)	1.000
Known CAD	6 (19)	8 (29)	10 (42)	0.183
Time between DSE and the event (months)	28 ± 3	25 ± 8	23 ± 8	0.382
Events	2 (6.5)	5 (18)	11 (46)	0.002

BMI: body mass index; CAD: coronary artery disease; DSE: dobutamine stress echocardiography. Measures expressed as number (percentage) or mean ± standard deviation.

### Echocardiographic and hemodynamic assessment

Of the echocardiographic variables assessed only at baseline, LVEF was preserved, while left ventricular mass index evidenced ventricular hypertrophy and did not differ between the groups. Regarding the echocardiographic and hemodynamic variables recorded at rest and during stress, the segmental contraction score index did not differ between the groups, and the frequency of DSE compatible with myocardial ischemia was low. Heart rate and double product did not differ; however, the groups differed in the number of patients attaining maximal HR predicted for age (Table 2).

**Table 2:** Echocardiographic and hemodynamic variables by group

Patients		Group I 31	Group II 28	Group III 24	p
**Ejection fraction (%)**					
	(Rest)	65 ± 7	67 ± 4	62 ± 9	0.019
**LVMI (g/m^2^)**					
	(Rest)	126 ± 29	130 ± 45	135 ± 37	0.670
**SCSI**					
	(Rest)	1.04 ± 0.15	1.02 ± 0.06	1.06 ± 0.21	0.086
	(Stress)	1.03 ± 0.09	1.02 ± 0.05	1.07 ± 0.24	0.949
Stress without ischemia		30 (96,8)	26 (92.9)	21 (87.5)	0.430
**HR (bpm)**					
	(Rest)	68 ± 12	68 ± 11	74 ± 12	0.096
	(Stress)	149 ±11	147 ± 13	147 ± 11	0.677
Maximal HR achieved		2 (6,5)	6 (21)	10 (42)	0.007
**Double product (mmHg.bpm)**					
	(Rest)	8548 ± 2010	8749 ± 2159	9681 ± 2020	0.107
	(Stress)	22108 ± 2896	22700 ± 3449	22215 ± 2833	0.742
**PDV (cm/s)**					
	(Rest)	24 ± 5	28 ± 6	38 ± 8	<0.0001
	(Stress)	60 ± 16	68 ± 15	65 ± 17	0.143
**HR at PDV**					
	(Stress)	105 ± 16	135 ± 14	132 ± 17	<0.0001
CFVR		2,53 ± 0,60	2.50 ± 0.57	1.7 ± 0.24	<0.0001

LVMI: left ventricular mass index; SCSI: segmental contraction score index; HR: heart rate; double product: systolic blood pressure x heart rate; PDV: peak diastolic velocity; CFVR: coronary flow velocity reserve. Measures expressed as number (percentage) or mean ± standard deviation.

On Doppler assessment of LAD, the groups differed regarding the HR during PDV recording at rest, as well as the PDV values at rest. However, during stress, PDV did not differ, resulting in different CFVR values in the groups (Table 2). When comparing those different echocardiographic and hemodynamic variables, in Group III, PDV at rest was higher and CFVR was lower than in the other two groups; however, those variables did not differ when comparing Groups I and II. The HR during stress on PDV recording in Group I was lower than in Groups II and III, compatible with this study protocol. The LVEF differed between Groups II and III (Table 3).

**Table 3:** Comparison of the different variables by group

	Group I	Group II	Group III	p*	p**	p***
PDV (Rest)	24 ± 5	28 ± 6	38 ± 8	< 0.001	0.001	0.105
CFVR	2.53 ± 0.6	2.50 ± 0.6	1.69 ± 0.2	< 0.001	< 0.001	1.000
HR at PDV	105 ± 16	135 ± 14	132 ± 17	< 0.001	1.000	< 0.001
Ejection fraction	65 ± 7	67 ± 4	62 ± 9	0.072	0.023	1.000

PDV: peak diastolic velocity; CFVR: coronary flow velocity reserve; HR: heart rate.

p* (Group III vs I);

p** (Group III vs II);

p*** (Group II vs I).

**Figure 1:**
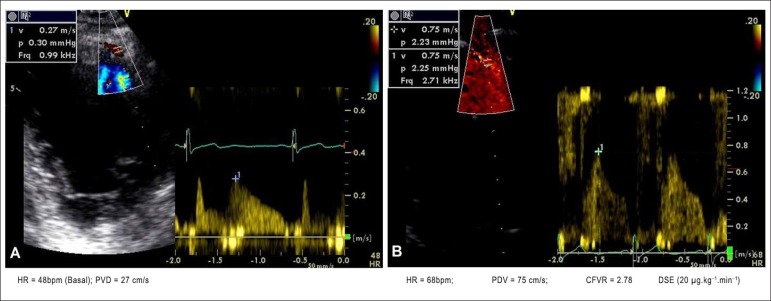
Male patient with target heart rate (HR) of 142 bpm. Figure 1A shows Doppler assessment in the left anterior descending coronary artery (LAD) at baseline. Figure 1B, during dobutamine stress echocardiography (DSE), dose = 20 µg.kg^-1^.min^-1^ and HR = 68 bpm, shows normal (=2.78) and early (obtained before achieving target HR) coronary flow velocity reserve (CFVR). Normal left ventricular contractility during the entire exam.

**Figure 2:**
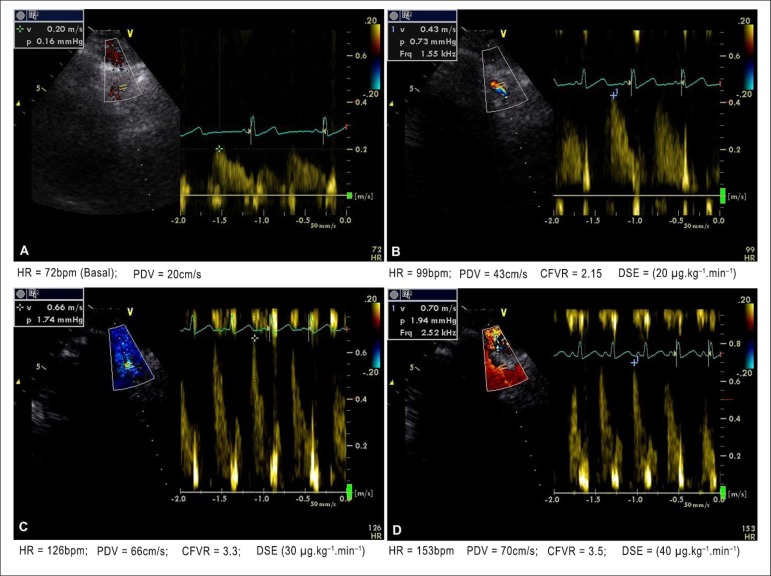
Male patient with target heart rate (HR) of 140 bpm. Figure 2A shows Doppler assessment in the left anterior descending coronary artery (LAD) at baseline. Figure 2B, during dobutamine stress echocardiography (DSE), dose = 20 µg.kg^-1^.min^-1^ and HR = 99 bpm, shows normal (2.15) and early coronary flow velocity reserve (CFVR), which increases progressively, even after reaching target HR (Figure 2D). Normal left ventricular contractility during the entire exam.

It is worth noting that, on several occasions, normal early CFVR could be obtained with the infusion of dobutamine at the dose of 20 *µ*g.kg-^1^.min-^1^, when HR was far below the submaximal HR calculated for the case (Figures 1 and 2).

### Presence or absence of ischemia during DSE and occurrence of events

Of all DSE performed, 6 (7.2%) were positive for myocardial ischemia, 1 in Group I, 2 in Group II and 3 in Group III, and events were observed in 4 of those 6 patients, with 1 coronary angiography without further intervention and 1 stent implantation in Group II and Group III. Considering all 83 patients studied, the mean time for occurrence of events was 17 ± 8 months. During the follow-up of 28 ± 4 months, events were observed in 18 (21.6%) patients as follows: 4 deaths (4.8%); 6 coronary interventions (7.2%); and 8 coronary angiographies without further intervention (9.6%). Considering all events, 6% (2/31) occurred in Group I, 18% (5/28) in Group II, and 46% (11/24) in Group III (Table 4).

**Table 4:** Distribution of the cases regarding the presence or absence of ischemia during dobutamine stress echocardiography (DSE) and the occurrence of events in the groups

Groups	Ischemia during DSE	Myocardial segment affected	Events	Mean time between DSE and event (months)
Group I (CFVR ≥ 2)	No	-	Death	26.5
	No	-	Coronary angiography	13.4
Group II (CFVR ≥ 2)	Yes	Septal	Coronary angiography	1.1
	Yes	Septal	Stent	15.3
	No	-	Stent	15.4
	No	-	Coronary angiography	12.5
	No	-	Death	3.1
Group III (CFVR < 2)	No	-	Stent	28
	No	-	Coronary angiography	8.3
	No	-	Stent	21
	No	-	Stent	14.8
	No	-	Death	7.2
	No	-	Coronary angiography	14.7
	Yes	Lateral	Stent	17
	No	-	Coronary angiography	7.9
	Yes	Inferior	Coronary angiography	24.3
	No	-	Death	19.8
	No	-	Coronary angiography	22.4

Normal coronary flow velocity reserve (CFVR ≥ 2) was obtained before (Group I) and after (Group II) reaching submaximal heart rate. Coronary angiography - hemodynamic study without further coronary intervention (angioplasty, stent or surgery).

Of the 8 coronary angiographies without further intervention, only 3 were performed within 1 year of follow-up (1 in Group II and 2 in Group III), while all interventions (stent implantation) were performed after 1 year of follow-up. Of the 4 deaths, 1 occurred in Group I (26.5 months after DSE) and was attributed to complications after myocardial revascularization surgery. The death in Group II (3 months after DSE) occurred during heart surgery to treat exacerbated mitral insufficiency (secondary to valve prolapse) and CAD. The other 2 deaths were observed in Group III: 1 simultaneous with pulmonary embolism; and 1 occurred 20 months after DSE during heart surgery for heart valve replacement in a patient with calcified coronary arteries. During the follow-up, we obtained no information allowing us to infer the diagnosis of the acute coronary insufficiency or of the hospitalization due to a cause other than those already cited (Table 4).

**Figure 3:**
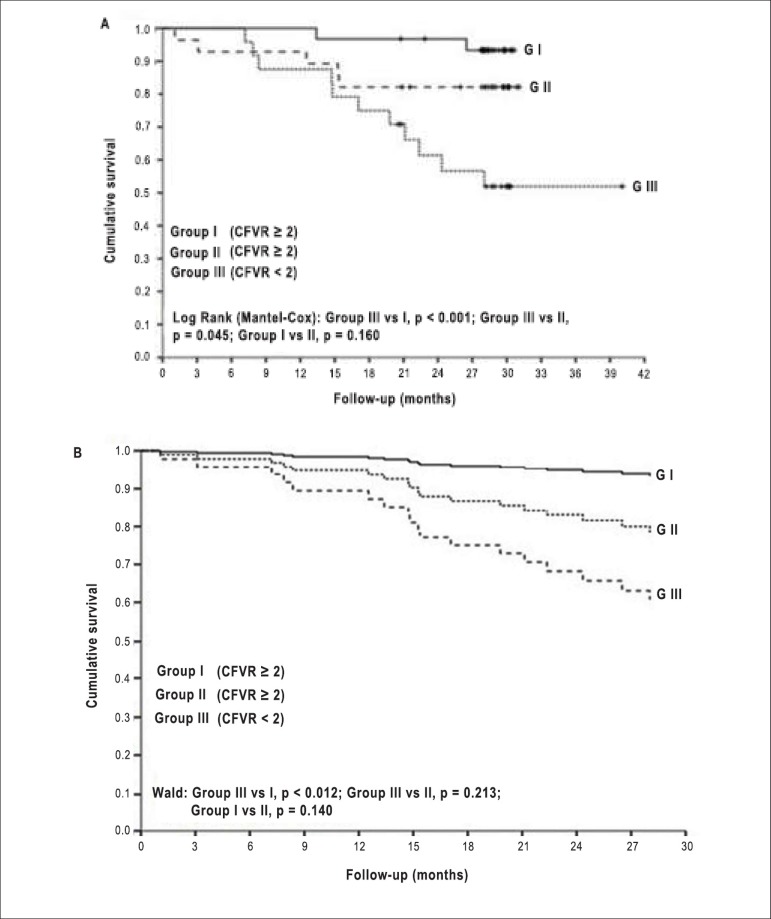
A) Kaplan-Meier regression analysis of event-free survival by group. Normal (= 2) coronary flow velocity reserve (CFVR) was obtained before (Group I) and after (Group II) reaching submaximal target heart rate. In Group III, CFVR was abnormal. Group III differed from Group I and Group II, but there was no difference between Group I and Group II. B) Survival for the event adjusted for age and ejection fraction, by use of Cox regression and Wald statistics. Group III and Group I remained different, and the better event-free survival was maintained only in Group I.

Regarding the Kaplan-Meier regression analysis of event-free survival, Groups I and II did not differ, and had a better outcome than Group III. However, after adjusting for age and LVEF, Group II did not differ from Group III, and the best event-free survival was maintained only in Group I when compared to Group III (Figure 3).

## Discussion

A negative pharmacologic stress echocardiography for ischemia associates with good prognosis and less need for myocardial revascularization. However, in both micro- and macrocirculation contexts, CFVR obtained in the LAD adds value to the information provided by stress echocardiography. Patients with exams showing normal myocardial contractility and normal CFVR in the LAD have mortality lower than 1% per year, while those with impaired contractility and abnormal CFVR have mortality greater than 10% per year. Even for octogenarians, RFVC is a strong and independent predictor of death and of myocardial infarction, mainly when contractility is preserved. Those results support the measurement of CFVR during pharmacologic stress echocardiography, favoring its incorporation into routine practice.^10,12-15,25,26^

In several studies assessing myocardial ischemia or risk stratification by use of transthoracic echocardiography, CFVR and contractile abnormality are evaluated by use of adenosine or dipyridamole, or patients are submitted to an additional stress with dobutamine to assess the induction of contractile abnormality. However, the vasodilator effect of dobutamine in the presence of preserved LVEF is comparable to that of adenosine, which is similar to that of dipyridamole. Because dobutamine is one of the most used drugs in stress echocardiography, it is worth noting the possibility of, in the same exam, having a consistent positive inotropic effect on the cardiac muscle and a proper coronary vasodilating effect to calculate CFVR.^6,12,13,15,20,21,27^

The CFVR obtained during DSE can anticipate the probable result of the exam regarding cardiac muscle contractility, so that a better reserve associates with better contractile response, regardless of whether CFVR is obtained early or late.^21,22^ However, the prognostic value of early CFVR has not been established in the literature, and this has motivated the present study.

The groups assessed in this study did not differ regarding age, sex, presence of hypertension, dyslipidemia, diabetes and known CAD, use of medications, and not even left ventricular mass index and baseline double product and HR, factors that could influence the measurement of baseline PDV, and, consequently, CFVR.^28^ However, to which extent pathological conditions, such as hypertension, diabetes and dyslipidemia, affect each individual cannot be established. Thus, Group III had higher baseline PDV, which could express predominance of an abnormal microvascular component over a possible epicardial coronary artery stenosis. However, regardless of which component (micro- or macrovascular) is more important, both can be related to worse prognosis.^10,12-15,25,27^

The recording of a slightly altered segmental contraction score index at rest and during stress might have resulted from the preserved LVEF and lack of ischemic response in a greater number of exams. During the study, 4 of the 6 patients with positive DSE for ischemia had events, which might have been expected by their attending physicians or might partially represent a bias. However, although 77 of the 83 patients (92.8%) could be considered of low risk because of their negative DSE, determining the expectation of good prognosis, the CFVR measure provided better risk stratification. Regarding the patients with negative DSE for ischemia and later submitted to intervention, progression of preexisting CAD might have occurred or there might have been a false-negative DSE for ischemia. An explanation for that could be the fact that maximal HR was not achieved.^29^

The hemodynamic studies were requested by the attending cardiologists, as were the further interventions, which were indicated based on the importance of the coronary artery stenosis. Most hemodynamic studies (11/14 - 79%) were performed after 1 year of follow-up. However, it is worth noting that, considering only patients with negative DSE for ischemia, in 80% (8/10) of those with hemodynamic study, that study was performed more than 1 year after the DSE, possibly expressing rather a clinical decision than a bias of the CFVR result previously informed. Those observations can suggest that the disclosure and recognition of the importance of the CFVR obtained on stress echocardiography is still limited, which could determine decisions rather related to the presence or absence of ischemia. Based on the research protocol, we do not interfere with the management of the attending cardiologists, but it is worth noting that only 1 hemodynamic study was requested for Group I patients.

The 4 deaths in this study occurred among patients with negative DSE for myocardial ischemia. In Group I, that event occurred after myocardial revascularization, which was performed 2 years after the exam. In Group II, the death occurred 3 months after the DSE, resulting from complications during surgery to repair acute mitral insufficiency in a patient with mitral valve prolapse. However, the death certificate available did not provide further information on the relevance of the CAD reported. In Group III, 1 death was attributed to pulmonary embolism, and the other death occurred in a patient who met no criteria for severe heart valve disease on DSE. The patient died 20 months after the exam, during heart surgery for heart valve replacement, when calcified coronary arteries were identified.

On the Kaplan-Meier regression analysis of event-free survival, the groups of patients with normal CFVR did not differ between themselves, and had better outcome than those with abnormal CFVR. However, after adjusting for age and LVEF, Group II did not differ from Group III, and the better event-free survival was maintained only for Group I.

The literature shows that the prognosis of patients with normal CFVR is better than that of patients with abnormal CFVR. However, in our study, the patients with preserved LVEF only had better prognosis in the presence of normal early CFVR.

Figure 1 shows that, with a HR of 68 bpm, normal early CFVR (= 2.78) could already be obtained, demonstrating the significant vasodilating effect of dobutamine infusion (20 *µ*g.kg^-1^.min^-1^). Figure 2 shows the higher baseline HR recording, but like the previous case, normal early CFVR (= 2.15) was also obtained in the second stage of DSE, with the simultaneous HR of 98 bpm. Those findings are in accordance with those by Takeuchi et al.,^21^ who have reported that patients with normal CFVR recorded in the intermediate stage of DSE (20 *µ*g.kg^-1^.min^-1^) belonged to the group that had no myocardial contractile abnormality next to the coronary artery assessed. In addition, Ahmari et al.^22^ have reported that patients who developed no ischemia had a better CFVR with the intermediate dose of dobutamine.

In our study, all patients with normal early CFVR maintained that normal condition during all stages of DSE, and none of them showed contractile impairment of the anterior wall. This suggests that, from the time normal early CFVR is achieved, continuing its recording is no longer necessary. In a study including only patients at low risk for CAD, Forte et al.^20^ have reported that, during DSE, 96% of the patients achieved normal CFVR before reaching submaximal target HR, and all of them had a negative exam for ischemia. In our study, the findings of Group I could result from a smaller impairment of the micro- and macrocirculation, which favors the attainment of normal early CFVR during DSE. However, further studies are necessary to confirm this hypothesis.

### Clinical implications

Attaining normal early CFVR in the LAD identifies patients with better prognosis. In addition, in that condition, the occurrence of contractile abnormality in the anterior wall during DSE is unlikely. In the exclusive context of contractile abnormality, normal early CFVR is particularly useful when the visualization of the anterior wall is hindered during a stage with higher HR, or even when maximal HR is not achieved, because the accuracy of DSE is lower in that circumstance.^29^

### Limitations

Despite the prospective nature of this study, some limitations apply. A larger sample and a longer follow-up could add more information. However, this study sample size and follow-up duration were similar to those of some studies here cited. To assess prognostic value, the CFVR was obtained through PDV recorded only in the LAD, but that condition has been validated and used in several studies cited in the present study. The most important limitation of this study is that we neither had complete access to the therapy used by the attending cardiologists, nor knew the reasons for choosing each patient's management, mainly regarding the coronary angiographies without further intervention.

## Conclusion

In patients with preserved LVEF and who completed the DSE, the normal CFVR obtained before achieving submaximal target HR associated with better prognosis. This study suggests that, after attaining normal early CFVR, continuing its recording is no longer necessary.
