# Erratum

**DOI:** 10.5935/abc.20170092

**Published:** 2017-06

August issue of 2012, vol. 99 (2), Suppl. 2, pages 1-28

In "First Brazilian Guidelines for Familial Hypercholesterolemia", consider Lottenberg AM as the correct form for the name of the author Ana Maria Lottenberg.

May issue of 2017, vol. 108 (5), pages. 417-426

In the Original Article "Prognostic Value of Coronary Flow Reserve Obtained on Dobutamine Stress Echocardiography and its Correlation with Target Heart Rate", pages 417-426, by authors José Sebastião de Abreu, Eduardo Arrais Rocha, Isadora Sucupira Machado, Isabelle Oliveira Parahyba, Thaís de Brito Rocha, Fernando José Villar Nogueira Paes, Tereza Cristina Pinheiro Diogenes, Marília Esther Benevides de Abreu, Ana Gardenia Liberato Ponte Farias, Marcia Maria Carneiro, José Nogueira Paes Junior, please be aware that the correct spelling for Isabelle O. Parahyba is Isabelle Oliveira Parahyba and Thais Brito Rocha is Thaís de Brito Rocha.

